# Qualitätssteigerung der Dokumentation zur Biologikatherapie der chronischen Rhinosinusitis durch digitale, strukturierte Befunderhebung und Indikationsstellung?

**DOI:** 10.1007/s00106-024-01488-x

**Published:** 2024-05-30

**Authors:** Jan Hagemann, Christopher Seifen, Laura Koll, Manuel Reissig, Barbara Leggewie, Berit Hackenberg, Julia Döge, Kai Helling, Sven Becker, Ludger Klimek, Christoph Matthias, Benjamin-Philipp Ernst

**Affiliations:** 1 Hals‑, Nasen‑, Ohrenklinik und Poliklinik, Universitätsmedizin Mainz, Langenbeckstraße 1, 55131 Mainz, Deutschland; 2 Klinik und Poliklinik für Hals-Nasen-Ohren-Heilkunde, Universitätsklinikum Bonn, Venusberg Campus 1, 53127 Bonn, Deutschland; 3 Universitätsklinik für Hals‑, Nasen- und Ohrenheilkunde, Universitätsklinikum Tübingen, Elfriede-Aulhorn-Straße 5, 72076 Tübingen, Deutschland; 4 Zentrum für Allergologie und Rhinologie, An den Quellen 10, 65183 Wiesbaden, Deutschland; 5 Klinik für Hals‑, Nasen‑, Ohrenheilkunde, Universitätsklinikum Frankfurt, Theodor-Stern-Kai 7, 60596 Frankfurt am Main, Deutschland

**Keywords:** Personalisierte Medizin, Elektronische Krankenakte, Gesundheitsbezogene Informationstechnologie, Datendokumentation, Digitales Gesundheitswesen, Personalized medicine, Electronic health records, Health information technology, Data reporting, Digital health

## Abstract

**Hintergrund:**

Biologika ergänzen durch gezielte, hemmende Mechanismen der Typ-2-Entzündung die Standardtherapie für unzureichend kontrollierte schwere Formen der chronischen Rhinosinusitis mit Nasenpolypen (CRSwNP). Trotz Standardisierung mithilfe papierbasierter Checklisten stellen Dokumentation von Anamnese und notwendigen Befunden zur Erfüllung aktueller Verordnungskriterien eine große Herausforderung für Ärzt:innen dar. Ziel der vorliegenden Studie war es, mithilfe von strukturierter Befunderhebung („*structured reporting*“, SR) die Qualität jener Dokumentation und den Therapieentscheidungsprozess effizienter zu gestalten. Als Vergleich dienten hierzu die bisher erhältlichen Papier-Checklisten.

**Methoden:**

Für diese Studie wurde ein inkrementelles Tool programmiert, um aktuelle Befunde zu erfassen und die Erfüllung der Indikationskriterien zu überprüfen. Das Tool wurde in puncto Vollständigkeit, Zeitaufwand und Lesbarkeit mit anderen Checklisten verglichen

**Ergebnisse:**

Für jede der 3 Dokumentationsmöglichkeiten wurden 20 Befunde erhoben und in die Analyse einbezogen. Die Dokumentation auf den papierbasierten Checklisten hatte einen vergleichbaren Informationsgehalt: 17,5 ± 5,1 bzw. 21,7 ± 7,6 von maximal 43 möglichen Punkten; *p* *>* *0*,05. Die Dokumentation mit der digitalen Anwendung führte zu einem signifikanten Anstieg des Informationsgehalts im Vergleich zu allen papierbasierten Dokumentationen. Die durchschnittliche Punktzahl betrug 38,25 ± 3,7 (88,9 % der Maximalpunktzahl; *p* < 0,001). Die Nutzerzufriedenheit war im Durchschnitt hoch (9,6/10). Die Nutzung der digitalen Anwendung war anfangs zeitaufwendiger, verringerte sich aber mit zunehmender Anzahl der dokumentierten Fälle erheblich.

**Schlussfolgerung:**

Die strukturierte Befundung mittels (Web‑)Apps könnte in Zukunft die papierbasierte Befundung zur Indikation einer Biologikatherapie bei CRSwNP-Patient:innen ersetzen und zusätzliche Vorteile in Bezug auf die Datenqualität und Nachvollziehbarkeit der Ergebnisse bieten. Das zukünftig steigende Dokumentationsvolumen, die fortschreitende Digitalisierung und die Möglichkeit der Vernetzung zwischen einzelnen Zentren machen die Einführung einer App in naher Zukunft wahrscheinlich und wirtschaftlich.

**Zusatzmaterial online:**

Zusätzliche Informationen sind in der Online-Version dieses Artikels (10.1007/s00106-024-01488-x) enthalten.

## Abstract (en)

**Background:**

With targeted inhibition of type 2 inflammation, biologics represent the standard add-on therapy for inadequately controlled severe forms of chronic rhinosinusitis with nasal polyps (CRSwNP). Despite standardization with paper-based checklists, the documentation of medical history and current findings pertinent to indication criteria are a significant challenge for physicians. Through development of an application based on structured reporting, the current study aimed to improve documentation quality and simplify the decision-making process. Previously available paper checklists served as a comparison.

**Methods:**

For this study, a digital incremental tool was programmed to record current findings and check for fulfilment of indication criteria. The tool was compared with other checklists in terms of completeness, time required, and readability.

**Results:**

A total of 20 findings were collected for each of the three documentation options and included in the analysis. Documentation with the two paper-based checklists had comparable information content: 17.5 ± 5.1/21.7 ± 7.6 points out of a maximum of 43 points; *p* > 0.05. Documentation using the digital application led to a significant increase in information content compared to all paper-based documentation. The average score was 38.25 ± 3.7 (88.9% of maximum; *p* < 0.001). On average, user satisfaction was high (9.6/10). Use of the digital application was initially more time consuming, but as more cases were documented, the time taken improved significantly.

**Conclusion:**

In the future, structured reporting using apps could replace paper-based reporting for the indication of biologic therapy in CRSwNP patients and offer additional benefits in terms of data quality and traceability of results. The increasing volume of documentation in the future, the progress of digitalization, and the possibility of networking between individual centers make introduction of the app in the near future both likely and economical.

## Biologikaverordnung

Seit mehreren Jahren stellen Biologika mit gezielter Hemmwirkung gegen verschiedene Mechanismen der Typ-2-Inflammation die Standard-Zusatztherapie für unzureichend kontrollierte, schwere Formen der chronischen Rhinosinusitis mit Polyposis nasi (CRSwNP) dar [1, 2]. Die Präparate werden nicht nur für die CRSwNP, sondern auch für andere Erkrankungen aus dem Kreis der Typ-2-Inflammation eingesetzt und haben zu einem Paradigmenwechsel in der Therapie jener schweren, chronischen Entzündungen geführt [3]. Die Verordnungssituation der Biologika wurde in den vergangenen Jahren durch Implementierung standardisierter Abläufe, Veröffentlichung nationaler und europäischer Leitlinien sowie überzeugender Studiendaten zu Wirksamkeit und Sicherheit vereinfacht. Dies führt insgesamt zu einer Zunahme der Verordnung von Biologika, sowohl durch niedergelassene Hals-, Nasen-, Ohren(HNO)-Ärzt:innen als auch durch spezialisierte rhinologische Zentren. Die Dokumentation von aktuellen Befunden, Krankenvorgeschichte, Erfüllung der Indikationskriterien, Veränderung der Krankheitskontrolle unter Therapie und Verträglichkeit wird durch die Leitlinien immer wieder hervorgehoben und nicht zuletzt aus medikolegalen Gesichtspunkten explizit empfohlen [2, 4]. Dies scheint der wichtigste Faktor zur Vermeidung häufig befürchteter und zuletzt zugenommener Regressforderungen durch die Krankenkassen zu sein [5]. Die Dokumentation stellt die Behandler:innen aufgrund einer Vielzahl unterschiedlicher Abläufe in den einzelnen Zentren, Personalmangel und Verdichtung der Arbeitsintensität vor erhebliche Herausforderungen.

Die strukturierte, standardisierte Befunderhebung („*structured reporting*“; SR) für die Behandlung der CRSwNP wird aktuell nicht flächendeckend angewendet [3]. Das Konzept findet in vielen Fachbereichen Anwendung und wird im Hinblick auf verbesserte intra- und interdisziplinäre Kommunikation und Therapieentscheidungen durch zahlreiche Studien beworben [6–8]. Neben einer vereinheitlichten Terminologie für alle Anwender:innen führt SR mitunter zu vollständigerer Dokumentation und möglicherweise auch zu Zeitersparnis im Vergleich mit konventioneller, nichtstandardisierter Befunderhebung („*conventional reporting*“; CR). Gerade die Bemühungen zur Digitalisierung machen die Weiterentwicklung digitaler Tools unter Anwendung von SR dringend notwendig, um gemischte oder doppelte Dokumentation zu vermeiden und die Therapie und ihre Dokumentation auf eine zukunftsfähige Basis zu stellen.

Aktuell sind sich gerade bei der Frage der Entscheidung für oder gegen eine Biologikatherapie viele Behandler:innen unsicher. Checklisten als Hilfestellung für die Indikation bieten aber nur bedingt die Möglichkeit, aktuelle Befunde übersichtlich voranzustellen, sodass der Dokumentationsaufwand durch die Verwendung von Checklisten eher wächst. Die aktuelle Vielzahl der im deutschen Gesundheitssystem verwendeten Checklisten und Dokumentationsbögen (u.a. Ärzteverband Deutscher Allergologen e. V., AeDA; Deutsche Gesellschaft für Hals‑, Nasen‑, Ohren-Heilkunde; DGHNO; interne Checkliste der Universitätsmedizin [UM] Mainz; Sk2-Leitlinie der DGHNO/Deutschen Gesellschaft für Allgemeinmedizin und Familienmedizin [DEGAM]) war für die Autor:innen der Grund zur Entwicklung einer digitalen, inkrementellen Befunderhebung und Checkliste zur Indikationsstellung und diese mit Blick auf Vollständigkeit, Zufriedenheit der Anwender:innen und Zeitaufwand zu untersuchen. Ziel der Studie war es, dieses digitale Tool als eine Art digitale „Assistenz“ für die gesamte Spanne von Screening, Befunddokumentation, Indikationsstellung und Verlaufskontrolle zu verwenden und in der Anwendung zu erproben.

## Methoden

### Entwicklung der digitalen Befundmaske und Checkliste

**Abb. 1:**
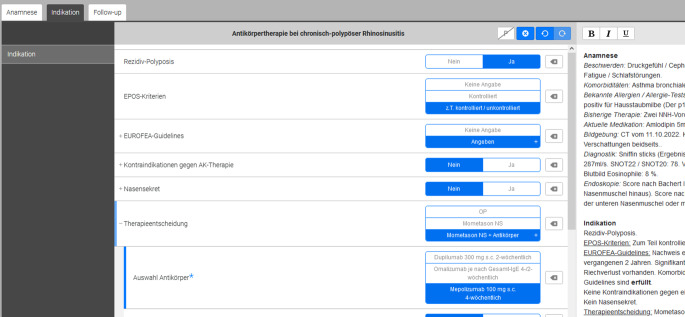
Exemplarischer Screenshot aus der digitalen App. Anamnese, Indikationskriterien und Follow-up getrennt zu bearbeiten. Parallel erstellter Fließtext (rechte Bildseite). (©Smart Reporting GmbH, München, Deutschland)

Für diese Studie wurde zunächst ein digitales inkrementelles Tool für Zwecke der Befunderhebung und Erfüllung bestimmter Kriterien programmiert (Abb. 1). Hierbei wurde wie schon in anderen Studien das „*frame work*“ der Fa. Smart Reporting GmbH, München, Deutschland, benutzt [9]. Die Frage, aus welchen Befunden die Dokumentation zur Beurteilung von Patient:innen mit schwerer CRSwNP bestehen sollte, hat in den vergangenen Jahrzehnten einen dynamischen Prozess durchlaufen. International besteht in der Literatur große Kongruenz in den folgenden Aspekten: Es sollten in möglichst reproduzierbarer und nachvollziehbarer Form sowohl subjektive als auch objektive Parameter der Erkrankungsaktivität und aktuellen Beschwerden festgehalten werden [3, 10]. Zudem gilt es Komorbiditäten, bisherige Behandlungen und die aktuelle Medikation festzuhalten. Folgende Parameter wurden in die digitale Maske integriert: aktuelle Symptome, Komorbiditäten, bisherige und aktuelle Therapie (jeweils Auswahl/Freitext), rhinologisch-allergologische Befunde, Bildgebungen, Körpergewicht, Ergebnisse psychophysischer Riechdiagnostik, Rhinomanometrie, Differenzialblutbild, Fragebögen zur Messung der krankheitsbezogenen Lebensqualität und visuelle Analogskalen zu Riechfunktion und globaler Erkrankungsintensität. Zur Frage der Indikationsstellung und Therapieentscheidung samt Präparatewahl wurden die im deutschen Gesundheitssystem gültigen Voraussetzungen zur Verordnung von Biologika angewandt [1]. Kontraindikationen mussten aktiv verneint werden. Zur Dokumentation von Befunden und der Therapieentscheidung im Rahmen eines *Follow-up* (in dieser Studie 16–24 Wochen) wurden dieselben Parameter sowie mögliche Nebenwirkungen erneut abgefragt und eine aktive Evaluation der aktuellen Situation zur Frage der Therapiefortsetzung/-veränderung gefordert.

Die digitale Befunderhebung mit der Infrastruktur der Fa. Smart Reporting GmbH, München, Deutschland, erfolgt webbrowserbasiert mittels eines mit der Maus (dem Finger bei Touchpad) anklickbaren, inkrementellen Entscheidungsbaums (Abb. 1).

### Gegenüberstellung der Befunderhebung und Indikationsstellung

Ein Personenkreis aus insgesamt 15 an der Universitätsmedizin Mainz (UM Mainz) und dem Universitätsklinikum Bonn (UKB) tätigen Ärzt:innen in verschiedenen Stadien ihrer HNO-ärztlichen Laufbahn wurde gebeten, retrospektiv eine möglichst große Zahl von Patient:innen mit bereits erfolgreich gestarteter *Add-on*-Biologikatherapie entweder anhand der neu entwickelten digitalen SR-Maske (Abb. 1) oder der Checklisten des/der AEDA/DGHNO/DEGAM/intern UM Mainz zu befunden und den Entscheidungsprozess für bzw. wider Biologika zu durchlaufen (Online-Zusatzmaterial S2). Die retrospektive Dokumentation zur rein internen, studienbezogenen Verwendung wurde im Rahmen der an der UM Mainz durchgeführten Biologika-Studie durch die hiesige Ethikkommission gestattet (Ethikkommission der Landesärztekammer Rheinland-Pfalz, #14337). Die ethischen Richtlinien und die Deklaration von Helsinki wurden respektiert. Die befragten Personen hatten unterschiedlich viel Erfahrung im Umgang mit den Checklisten und der Behandlung von Patient:innen mit schwerer CRSwNP und befanden sich zum Teil noch in der Weiterbildung zur/m Facharzt:in für HNO-Heilkunde. Die für die jeweilige Dokumentation benötigte Zeit wurde gemessen. Zudem wurden die befragten Personen gebeten, 7 Fragen bezüglich Praktikabilität und Zufriedenheit im Umgang mit der digitalen Lösung zu beantworten.

Die so entstandenen digitalen oder Papier-Befunde wurden anhand eines standardisierten Auswertungsbogens analysiert. Dieser prüft die erhobenen Befunde auf Leserlichkeit und Vollständigkeit (1 bis maximal 43 Punkte), wobei für jede zusätzlich dokumentierte Information und gute Lesbarkeit Pluspunkte vergeben wurde. Der Maximalwert von 43 Punkten stellt insofern einen Idealwert dar, der gleichbedeutend mit einer umfassenden, gut lesbaren Dokumentation der Krankengeschichte, der aktuellen Befunde und der Argumentation für oder wider eine *Add-on*-Biologikatherapie ist. Hierbei sei zu beachten, dass im medizinisch-wissenschaftlichen Diskurs diese Idealvorstellung ständigen Veränderungen und Weiterentwicklungen unterliegt und selbstverständlich bereits zur Veröffentlichung dieser Studie veraltet ist. Als standardisiertes Werkzeug zum Zeitpunkt der Studie eignet sie der Auswertungsbogen aber zum Vergleich der einzelnen Dokumentationsmethoden.

### Statistische Auswertung

Die statistische Auswertung erfolgte mit der Software GraphPad Prism Version 10 (Fa. GraphPad Software LLC, La Jolla, CA, USA). Es wurden ein *α* von 0,05 und ein *β* von 0,2 angenommen. Die statistische Beratung ergab eine Fallzahlschätzung von *n* = 42 (*n* = 14 für jede Gruppe) für einen erwarteten Effekt (Steigerung der Vollständigkeit) von mindestens 20 %.

## Ergebnisse

Von den 15 befragten Personen antworteten 12 auf die Anfrage zur Teilnahme an der Studie. Die Teilnehmenden HNO-Ärzt:innen waren zwischen 25 und 55 Jahren alt (Median: 32 Jahre). Insgesamt wurden jeweils 20 Befunde für jeden der 3 Dokumentationsmöglichkeiten erhoben und in die Auswertung miteinbezogen.

### Papierbasierte Dokumentation

**Abb. 2:**
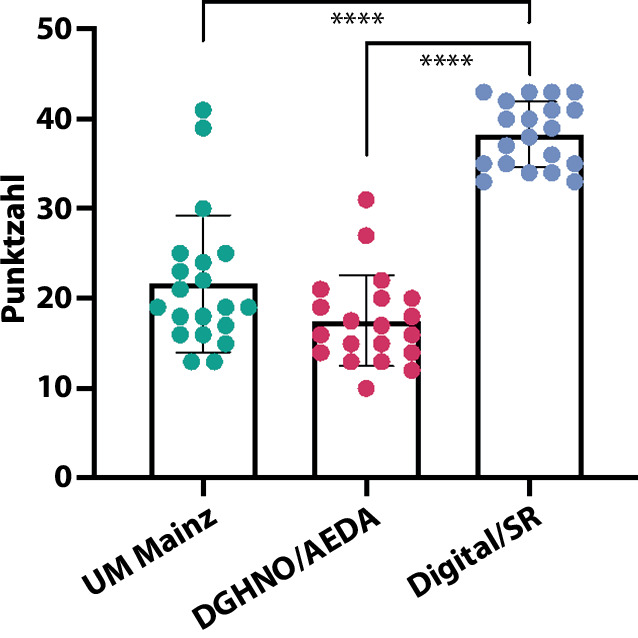
Ergebnisse zu Lesbarkeit und Informationsgehalt (Punktesystem mit maximal 43 Punkten). *Punkt *individuelle Werte, *Balken *Mittelwerte mit Standardabweichung (SD). *n* = 20/Gruppe. **** = *p* < 0,0001 in *1‑W-*ANOVA und *t*-test. *AeDA *Ärzteverband Deutscher Allergologen e. V.*; DGHNO *Deutsche Gesellschaft für Hals‑, Nasen‑, Ohren-Heilkunde; *SR* strukturierte Befunderhebung („*structured reporting*“); *UM Mainz *interne Checkliste der Universitätsmedizin Mainz

**Abb. 3:**
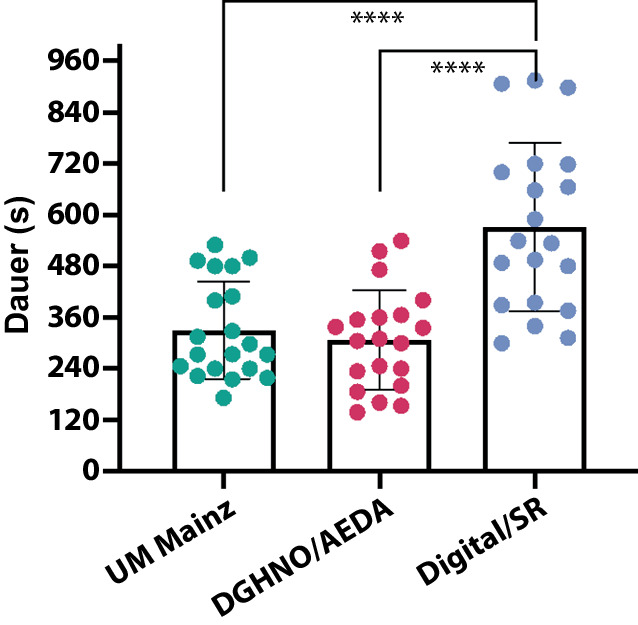
Ergebnisse zu benötigtem Zeitaufwand. In Sekunden. *Punkt *individuelle Werte, *Balken *Mittelwerte mit Standardabweichung (SD). *n* = 20/Gruppe. **** = *p* < 0,001 in *1‑W-ANOVA* und *t*-test. *AeDA *Ärzteverband Deutscher Allergologen e. V.*; DGHNO *Deutsche Gesellschaft für Hals‑, Nasen‑, Ohren-Heilkunde; *SR* strukturierte Befunderhebung („structured reporting“); *UM Mainz *interne Checkliste der Universitätsmedizin Mainz

Die Dokumentation mit den beiden papierbasierten Checklisten hatte vergleichbaren Informationsgehalt: Der von der DGHNO bzw. dem AeDA verbreitete Bogen erreichte im Schnitt 17,5 ± 5,1 von maximal 43 Punkten (40,1 %), der an der UM Mainz verwendete interne Bogen 21,7 ± 7,6 Punkte (50,4 %; *p* > 0,05; Abb. 2). Ebenso nicht signifikant unterschieden sich die beiden papierbasierten Bögen in Bezug auf den für das komplette Ausfüllen benötigten Zeitaufwand: Im Durchschnitt benötigten die Anwender:innen zwischen 5 und 6 min für das Herauslesen der Befunde aus den medizinischen Unterlagen der Patient:innen und das möglichst komplette Ausfüllen des Bogens (307 vs. 330 s für DGHNO/AeDA vs. UM Mainz; *p* > 0,05; Abb. 3). Die Lesbarkeit der Befunde war im Durchschnitt mit 2,8 Punkten nur beinahe mittelmäßig (Skala von 1 = schlecht bis 5 = sehr gut).

### Digitale Dokumentation

Die Dokumentation mithilfe der digitalen App und aufeinander aufbauendem Entscheidungsbaum führte im Vergleich zur papierbasierten Dokumentation zu einer deutlichen Steigerung des Informationsgehalts. Der durchschnittliche Punktwert lag bei 38,3 ± 3,7 von 43 möglichen Punkten (88,9 %, siehe Abb. 2). Sowohl im Einzelvergleich (*t*-test) als auch mittels einfaktorieller ANOVA war der Unterschied zu den papierbasierten Verfahren hochsignifikant (*p* < 0,001). Unerwarteterweise fiel jedoch auch der benötigte Zeitaufwand signifikant zuungunsten der digitalen Dokumentation aus. Im Durchschnitt benötigten die Personen in der vorliegenden Pilotstudie rund 75 % mehr Zeit für die digitale Dokumentation im Vergleich zur handschriftlichen (571 ± 196 s), wobei die Streubreite hier mit mehr als 3 min wesentlich höher ausfiel (Abb. 3). Während der Befunderstellung generierte die App einen Fließtext, der im Anschluss in andere Dokumentationssysteme übertragbar war (Online-Zusatzmaterial S1).

### Zufriedenheit

**Abb. 4:**
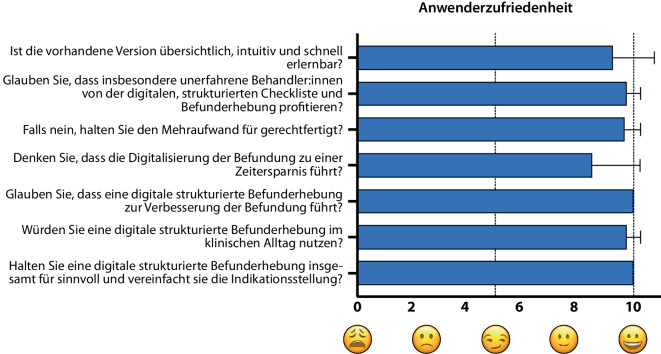
Zufriedenheit der Anwender:innen mit der digitalen Befunderhebung anhand von 7 Fragen. 0 = trifft nicht zu/unzufrieden bis 10 = trifft voll zu/voll zufrieden. *n* = 12

Dem in dieser Pilotstudie beobachteten hohen Zeitaufwand steht eine insgesamt hohe Zufriedenheit der Anwender:innen gegenüber (Abb. 4). In insgesamt 7 Fragen sollten die Anwender:innen nach Verwendung des digitalen Dokumentationssystems ihre Erfahrungen bewerten. Die Fragen wurden anhand von visuellen Analogskalen (Smileys + numerische Analogskala; 0 bis 10 = Bestnote) beantwortet. Die durchschnittliche Benotung lag zwischen 8,5 und 10 und damit im annähernd voll bzw. im voll zufriedenen Bereich (Abb. 3). Am höchsten wurden die Fragen zur Verbesserung der Befundqualität und Vereinfachung der Indikationsstellung bewertet, am niedrigsten die Frage zur Möglichkeit einer Zeitersparnis bei der Dokumentation. Aufgrund der beobachteten schnellen Verbesserung des benötigten Zeitaufwands über mehrere Anwendungen hinweg zeigt sich das Potenzial der digitalen Dokumentation erst nach mehreren Anwendungen.

## Diskussion

Die Add-on-Therapie der schweren, unzureichend kontrollierten CRSwNP mit verschiedenen Biologika gehört mittlerweile zum Versorgungsstandard im deutschen Gesundheitssystem [2]. Während sich die Studienlage zu Wirksamkeit, Sicherheit und Compliance stetig verbessert, ist die Auswirkung von Positionspapieren und Leitlinien auf die Versorgungssituation unzureichend untersucht. Unter Zunahme der Verschreibungen und damit steigender Gesundheitskosten wird auch die Datenqualität der Befunderhebung und Nachvollziehbarkeit der Indikationsstellung eine gesteigerte Rolle spielen [5]. Die hier gezeigte Studie stellt die Ergebnisse eines Pilotprojekts zur digitalen, inkrementellen Befunderhebung und Therapieentscheidung für die Biologikatherapie bei CRSwNP vor.

### Inkrementelle Befunderhebung

Die Ergebnisse der Studie und Befragungen der Teilnehmer:innen an der UM Mainz und dem UKB sprechen grundsätzlich für die Machbarkeit einer inkrementellen Befunderhebung und Therapieentscheidung unter Computerassistenz im Kontext einer Biologikabehandlung bei schwerer CRSwNP. Die Daten sprechen für eine verbesserte Datenqualität in Bezug auf Lesbarkeit und Vollständigkeit gegenüber den hier getesteten Versionen von papierbasierter Dokumentation. Die Zufriedenheit der Anwender:innen war im Durschnitt sehr gut (≈ 9,6 von 10 Punkten) und ermutigt die Autor:innen, die Entwicklung der Anwendung weiter voranzutreiben und eine Integration in die klinische Routine anzustreben. Diese Ergebnisse stehen in Einklang mit anderen wissenschaftlichen Publikationen, die unter Anwendung von SR eine höhere Datenqualität für zahlreiche diagnostische Modalitäten ergaben [6–9]. Die hohe Zufriedenheitsrate und Akzeptanz unter Anwender:innen lässt sich am ehesten durch intuitive Bedienbarkeit am ohnehin verwendeten Computer, inkrementellen Aufbau der App und Zeitersparnis beim Erstellen ausformulierter Befundberichte erklären.

**Abb. 5:**
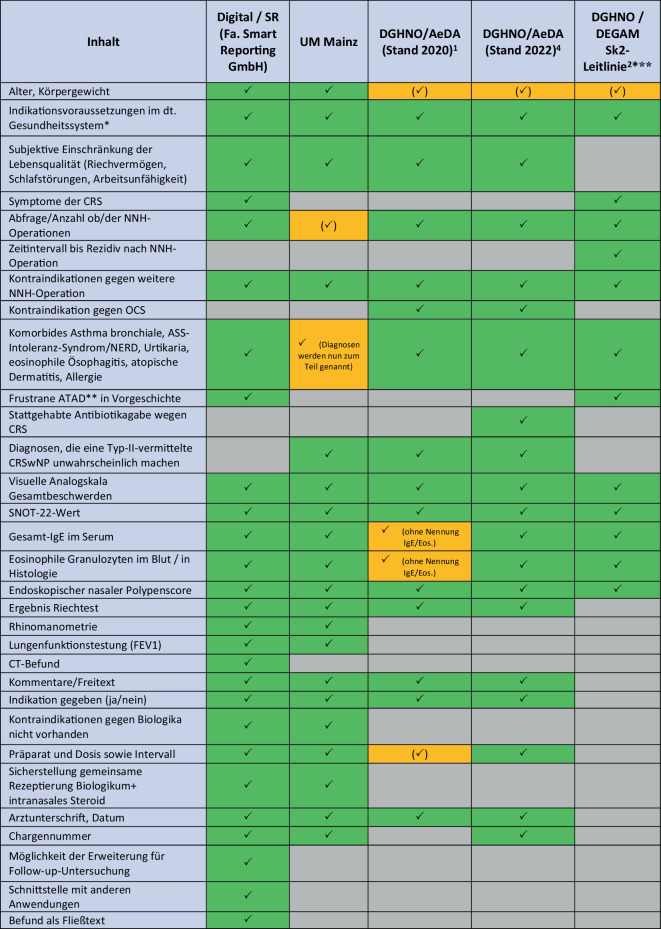
Unterschiede zwischen den Dokumentationsmöglichkeiten. * Diagnose, schwere Erkrankung, Z. n. oralen Kortikosteroiden (*OCS*) und/oder Nasennebenhöhlen(*NNH*)-Operation, fehlende Krankheitskontrolle unter topischen Steroiden; ** *ATAD*: adaptive Desaktivierung mit Acetylsalicylsäure (*ASS*); *** zum Zeitpunkt der Studie noch nicht verfügbar. *Grün *vorhanden. *Orange und Häkchen in Klammern *mit Einschränkung vorhanden. *Grau* nicht vorhanden. *AeDA *Ärzteverband Deutscher Allergologen e. V.*; CRS *chronische Rhinosinusitis*; CRSwNP *chronische Rhinosinusitis mit Polyposis nasi; *CT* Computertomographie; *DEGAM *Deutsche Gesellschaft für Allgemeinmedizin und Familienmedizin*;* *DGHNO *Deutsche Gesellschaft für Hals‑, Nasen‑, Ohren-Heilkunde; *FEV1* forciertes exspiratorisches Volumen; *IgE* Immunglobulin E; *NERD* ASS-Intoleranz-Syndrom („NSAID-exacerbated respiratory disease“); *SNOT* Sino-Nasal Outcome Test; *SR* strukturierte Befunderhebung („*structured reporting*“; Fa. Smart Reporting GmbH, München, Deutschland); *UM Mainz *interne Checkliste der Universitätsmedizin Mainz

Die Erstellung und Verbreitung von Checklisten als Begleitung für die Indikationsstellung einer Biologikatherapie hat die Verordnungssituation in Deutschland stark positiv beeinflusst [1, 4]. Sie dienen den Behandler:innen nicht nur als Entscheidungshilfe zur Frage der Notwendigkeit und Rechtfertigung einer *Add-on*-Biologikatherapie, sondern dienen darüber hinaus der Dokumentationen von u. U. schwerer Erkrankung, Vorgeschichte, Krankheitsaktivität und -belastung. Somit stellen sie eine Rechtfertigung für verordnungstechnische Rückfragen von Krankenkassen und anderen Überprüfungsorganen dar und wurden in Regressforderungen auch als solche bereits akzeptiert. Eine Schwierigkeit ergibt sich aus der aktuellen Vielzahl verschiedener Versionen, die sich untereinander maßgeblich unterscheiden (Abb. 5). Je nach verwendeter Version entstehen so wesentliche Dokumentationslücken in Bezug auf Krankenvorgeschichte oder Komorbiditäten. Die Checkliste der Sk2-Leitlinie verzichtet sogar gänzlich auf ein Kapitel zur Indikationsstellung und Präparatewahl [2]. Dies führt zwangsläufig zu doppelter und verschachtelter Dokumentation mit der restlichen Krankenakte und zu mehr Zeitaufwand bei Dateneingabe und Recherche.

Die Freiheit zur Wahl des Dokuments erschwerte sowohl die longitudinale Vergleichbarkeit als auch die Vergleichbarkeit zwischen verschiedenen Behandler:innen. Auch sind akademische Fragestellungen nur sehr schwer mit sinnvollem Zeitaufwand zu bewerkstelligen. Eine Integration in heutzutage meist verwendete digitale Krankenakten ist zudem nur mittels Scan möglich, sodass die Informationen nicht in bearbeitbarer Textform in der Akte erscheinen. Im Kontrast dazu lässt sich das von der Arbeitsgruppe der Autoren entwickelte und innerhalb dieser Studie erprobte digitale Tool aktualisieren, man kann einen Text sowohl exportieren als auch übersetzen und für andere technische Schnittstellen bereitstellen, wie z. B. für die in naher Zukunft geplante nationale Registerstudie der DGHNO in Zusammenarbeit mit dem AeDA.

### Integration in vorhandene Informationssysteme

Der in der vorliegenden Pilotstudie zur digitalen Dokumentation benötigte Zeitaufwand war im Durchschnitt signifikant größer im Vergleich zu den papierbasierten Methoden. In anderen Studien zu SR ließ sich hingegen eine Zeitersparnis bei Verwendung digitaler Anwendungen feststellen [11–13]. Die hier beobachteten Ergebnisse sind insofern zu relativieren, als dass erstens das Heraussuchen der Befunde aus der Krankenakte gerade wegen der uneinheitlichen, verschachtelten Dokumentation enorm zeitaufwendig ist. Die infrastrukturellen Probleme der aktuellen medizinischen Dokumentation an Universitätskliniken in Deutschland kommen hier vollumfänglich zum Tragen. Das hier entwickelte digitale Tool ist jedoch nicht für den retrospektiven, sondern alltäglichen klinischen Einsatz gedacht, sodass gerade erhobene Befunde direkt in den Entscheidungsbaum eingetragen werden können. Die Integration der SR-Anwendung in bereits vorhandene Informationssysteme wäre der nächste logische Entwicklungsschritt. Zweitens lässt sich aus der Abb. 5 schlussfolgern, dass wesentlich mehr Informationsgehalt im digitalen Tool möglich ist, was zukünftig doppelte Dokumentation ersparen wird. Die Zeit, die für die Ausformulierung in Fließtext nach Ausfüllen einer Papier-Checkliste benötigt wird, wurde in diesem Fall nicht gestoppt und ausgewertet. Drittens ist mit der Einführung einer neuen Maßnahme in der klinischen Routine immer auch mit einer initialen, transienten Verlängerung des Ablaufs zu rechnen [14]. Exemplarisch sei an dieser Stelle gezeigt, dass einer der Anwender:innen höheren Alters – was grundsätzlich die Eignung über alle Altersklassen hinweg beweist – für den ersten digitalen Befund mehr als doppelt so viel Zeit wie für den dritten Befund benötigte (899 s → 700 s → 312 s). Die Autoren vermuten abschließend, dass mit Investition in eine vorübergehende, kurze Gewöhnungsphase eine im Vergleich zur Papierversion vom Aufwand vergleichbare, aber qualitativ hochwertigere Dokumentation möglich ist, die die Behandler:innen gleichermaßen in Dokumentation und Entscheidungsfindung unterstützen könnte.

## Fazit für die Praxis

- Im Vergleich mit Checklisten vom Ärzteverband Deutscher Allergologen e. V. (AeDA), der Deutschen Gesellschaft für Hals-Nasen-Ohren-Heilkunde e. V. (DGHNO) und einer klinikinternen Checkliste konnte die Vollständigkeit und Benutzerzufriedenheit von Indikationsstellungen zur Biologikatherapie durch den Einsatz von „*structured reporting*“ signifikant gesteigert werden.
- Die Anforderungen an die Verordnung von Biologika im deutschen Gesundheitssystem, die Indikationsstellung und Therapieentscheidung, einschließlich der Wahl des Biologikums, können mittels „*structured reporting*“ im klinischen Alltag effektiv und nachvollziehbar dokumentiert werden.
- „*Structured reporting*“ mithilfe von Apps könnte in Zukunft die papierbasierte Befunderhebung zur Indikationsstellung einer Biologika-Therapie bei Patient:innen mit chronischer Rhinosinusitis mit Polyposis nasi (CRSwNP) ersetzen und bietet dabei zusätzliche Vorteile in Bezug auf Datenqualität und Nachvollziehbarkeit der Befunde.
- Die in Zukunft umfangreicher werdende Dokumentation, die voranschreitende Digitalisierung und Möglichkeit der Vernetzung zwischen einzelnen Zentren machen eine baldige Einführung der App in naher Zukunft wahrscheinlich und ökonomisch sinnvoll.

## Supplementary Information

SM 1: Beispiel eines automatisch erstellten Fließtextes nach Befundeingabe in die App

ESM 2: Indikationsstellung und Verlaufskontrolle der Biological-Therapie bei Polyposis nasi
