# Supplementary material for: Enhanced quality of documentation for biologic therapy of chronic rhinosinusitis through structured digital reporting and indication?
Source: HNO. 2024 May 30;73(2):103–10. [Article in German] doi: 10.1007/s00106-024-01488-x (PMC11772524; doi:10.1007/s00106-024-01488-x)
Supplement: Supplementary file 1 — SM 1: Beispiel eines automatisch erstellten Fließtextes nach Befundeingabe in die App [file 106_2024_1488_MOESM1_ESM.pdf]

Supplemental Material

Steigert die digitale, strukturierte Befunderhebung und Indikationsstellung die Qualität der Dokumentation zur Biologika-Therapie der Chronischen Rhinosinusitis?

Jan Hagemann, Benjamin Philipp Ernst, et al.

Supplemental Dokument 1: Beispiel eines automatisch erstellten Fließtextes nach Befundeingabe in die App. Dieser lässt sich zur Vermeidung von doppelter Dokumentation problemlos in andere Informationssysteme übernehmen.

Anamnese

Beschwerden: Druckgefühl / Cephalgie. Post-nasal drip. Hyposmie. Rhinorrhoe. Obstruktion. Fatigue / Schlafstörungen.
Komorbiditäten: Asthma bronchiale (FEV1 vom 01.07.2023: 2,1 L). Keine Analgetika-Intoleranz.
Bekannte Allergien / Allergie-Tests: Gesamt-IgE: 256 UI/ml. Prick-Test / nasaler Provokationstest: positiv für Hausstaubmilbe (Der p1).
Bisherige Therapie: Zwei NNH-Voroperationen. Cortison NS und SABA.
Aktuelle Medikation: Amlodipin 5mg 1-0-1.
Bildgebung: CT vom 11.10.2022. Keine seitengetrennte Verschattung. Pansinunasale Verschattungen beidseits..
Diagnostik: Sniffin sticks (Ergebnis: 6/16). Rhinomanometrie (Flow Gesamtnase vor Privin): 287ml/s. SNOT22 / SNOT20: 78. VAS Obstruktion: 8. VAS Riechvermögen: 1. Differenzial-Blutbild Eosinophile: 8 %.
Endoskopie: Score nach Bachert linksseitig: 2 (Polypen bis über den Unterrand der mittleren Nasenmuschel hinaus). Score nach Bachert rechtsseitig: 3 (Polypen erreichen den Unterrand der unteren Nasenmuschel oder medial der mittleren Nasenmuschel). **Gesamtscore: 5.**

Indikation

Rezidiv-Polyposis.
<u>EPOS-Kriterien:</u> Zum Teil kontrolliert / unkontrolliert.
<u>EUROFEA-Guidelines:</u> Nachweis einer Typ-II-Entzündung. Einnahme oraler Steroide in den vergangenen 2 Jahren. Signifikante Einschränkung der Lebensqualität. Signifikanter Riechverlust vorhanden. Komorbides Asthma bronchiale. Z.n. NNH-OP. Die EUROFEA-Guidelines sind **erfüllt**.
Keine Kontraindikationen gegen eine Antikörpertherapie..
<u>Therapieentscheidung:</u> Mometason NS + Antikörper.Mepolizumab 100 mg s.c. 4-wöchentlich. Rezeptierung Dupilumab + Mometason NS erfolgt. 1. Gabe erfolgt am 28.11.2023 (Chargennummer: ABCDEF99), keine Gegenreaktion.

Follow-up

<u>Follow-up (4-6 Monate)</u>
Endoskopie: Score nach Bachert linksseitig: 1 (Kleine Polypen im mittleren Nasengang, die nicht den Unterrand der mittleren Nasenmuschel überschreiten). Score nach Bachert rechtsseitig: 1 (Kleine Polypen im mittleren Nasengang, die nicht den Unterrand der mittleren Nasenmuschel überschreiten). **Gesamtscore: 2.**
Diagnostik: Sniffin sticks (Ergebnis: 9/16). Rhinomanometrie (Flow Gesamtnase vor Privin): 315. SNOT22 / SNOT20: 27. VAS Obstruktion: 3. VAS Riechvermögen: 7. Differenzial-Blutbild Eosinophile: 2 %.
Nebenwirkungen: Keine .
Evaluation: Reduktion der Polypen. Reduktion der oralen Steroide. Verbesserung der Lebensqualität / der subjektiven Krankheitsaktivität. Verbesserung des Geruchssinns. Reduktion der Symptome der Komorbiditäten.
Therapiefortsetzung.

<u>Follow-up (12 Monate)</u>
Endoskopie: Score nach Bachert linksseitig: 0 (Keine Polypen). Score nach Bachert rechtsseitig: 1 (Kleine Polypen im mittleren Nasengang, die nicht den Unterrand der mittleren Nasenmuschel überschreiten).
Gesamtscore: 1.
Diagnostik: Sniffin sticks (Ergebnis: 8/16). Rhinomanometrie (Flow Gesamtnase vor Privin): 423. SNOT22 / SNOT20: 18. VAS Obstruktion: 2. VAS Riechvermögen: 8. Differenzial-Blutbild Eosinophile: 2 %.
Nebenwirkungen: Keine.
Evaluation: Reduktion der Polypen. Reduktion der oralen Steroide. Verbesserung der Lebensqualität / der subjektiven Krankheitsaktivität. Verbesserung des Geruchssinns. Reduktion der Symptome der Komorbiditäten.
Therapiefortsetzung.
