# Supplementary material for: Enhanced quality of documentation for biologic therapy of chronic rhinosinusitis through structured digital reporting and indication?
Source: HNO. 2024 May 30;73(2):103–10. [Article in German] doi: 10.1007/s00106-024-01488-x (PMC11772524; doi:10.1007/s00106-024-01488-x)
Supplement: Supplementary file 2 — ESM 2: Indikationsstellung und Verlaufskontrolle der Biological-Therapie bei Polyposis nasi [file 106_2024_1488_MOESM2_ESM.pdf]

Indikationsstellung und Verlaufskontrolle der

Biological-Therapie bei Polyposis nasi

Name, Geburtsdatum:

Körpergewicht [kg]:

Patient/in >18 Jahre mit nicht ausreichend kontrollierter Polyposis nasi (CRSwNP) bei Z.n. NNH-Operation/en und/oder Z.n. oraler Cortison-Therapie und/oder bei OP-Kontraindikationen	✓
Die operative und konservative Therapie sind ausgereizt und andere Ursachen für Beschwerden / CT-Befunde als Typ-II Entzündung werden nicht vermutet	✓

Beratung des/der Patient/in zu Therapieoptionen (konservativ, operativ, Biologicals)

Der grobe Ablauf einer Biologica-Therapie sollte erläutert werden (s.c.-Gaben alle 2-4 Wochen, Erfolgskontrolle nach 4 Monaten)

Polypenscore (nach Bachert et al)	Siehe Seite 3, Summe bds.	
SNOT-20 GAV	Siehe Seite 4, Punktzahl	
VAS Nasenobstruktion / Geruch	Siehe Seite 4, Punktzahl	
Geruchstest Sniffin Sticks 16 Items	Punktzahl	
Rhinomanometrie (ohne Privin ausreichend)	Gesamtnase vor Privin ml/s	
Blutentnahme (orale Steroide absetzen!)	Diff-BB (EDTA)<br>Creatinin, Leberwerte (Li-Heparin)<br>Gesamt-IgE (Serum Braun)	
Bei komorbidem Asthma bronchiale: Lufu	FEV1	

Vorstellung des Falls in der Indikationsrunde (Donnerstags, 8 Uhr Bibliothek 3. Stock)

Einbestellung über Back Office oder persönlich (Sprechstunde Dienstags, Poli-Kabine 3)

Präparatewahl	**Dupilumab** 300 mg s.c. 2W	
	**Omalizumab** gewichts- und IgE-adaptiert s.c. 2W/4W	
	**Mepolizumab** 100 mg s.c. 4W	
Keine Kontraindikationen lt. Fachinformation, schriftl. Aufklärung		
Rezeptierung Präparat + Mometason NS:<br>- Erstverordnung Dupilumab / Mepolizumab nur N1 (2 Spritzen)<br>- Omalizumab 75 mg und 150 mg je nach indiv. Bedarf		
**Name, Datum:**		
Notizen / zu beachten vor Therapiebeginn		

AeDA Ärzteverband Deutscher Allergologen e.V.

Indikationsstellung zur Therapie der chronischen RhinoSinusitis mit Nasenpolypen (CRSwNP; Polyposis nasi) mit Biologika[1]

Nach: Klimek L. et al.: Positionspapier Anwendung von Biologika bei chronischer RhinoSinusitis mit Polyposis nasi (CRSwNP) im deutschen Gesundheitssystem von AeDA und DGHNOKHC, Laryngo-Rhino-Otol 2020; 99: 1–17

Allgemeine Daten:

Name: ………………………………………………… Vorname: ………………….……..…………… Geburtsdatum: …. …. ….…..

Indikation (ICD-10): …☐ J33.1… ☐ J33.8 ☐ J33.9… CRSwNP besteht seit: …………………………………..
(Mehrfachnennung möglich)

Eignungskriterien:

Gemäß des Positionspapiers ***Anwendung von Biologika bei chronischer RhinoSinusitis mit Polyposis nasi (CRSwNP) im deutschen Gesundheitssystem***1 besteht die Indikation zur Behandlung mit Biologika für Patienten, bei denen nachfolgende Kriterien erfüllt sind:

Alter: ≥ 18 Jahre ☐ Nein ☐ Ja
Diagnose: schwere CRSwNP ☐ Nein ☐ Ja
Aktuelle Behandlung mit nasalen GKS: ☐ Nein ☐ Ja
Einschränkung der Lebensqualität (QoL): ☐ Nein ☐ Ja, ggfls. SNOT-22 Score: ……….
Vorbehandlung mit systemischen GKS und/oder vorausgehende NNH-OP: ☐ Nein ☐ Ja

(Indikationsstellung nur, wenn 5mal „Ja" angekreuzt wurde)

Spezifizierung der Eignungskriterien:

Vorbehandlung mit systemischen GKS: ☐ Nein ☐ Ja
ca. …… mal in den letzten 5 Jahren, zuletzt: ………… Wirkstoff: ……………………………, Dosis mg: ………………. über …... Tage
NNH-Operationen: ☐ Nein ☐ Ja, insgesamt: … mal, davon in den letzten 10 Jahren: …. mal, zuletzt: ….….

Zusätzliche Klinische Eignungskriterien:

Nachweis/Hinweis auf eine zugrundeliegende Typ-2-Inflammation: ☐ Nein ☐ Ja
Nasaler Polypen-Score ≥ 4 von 8 (NPS: ………..): ☐ Nein ☐ Ja
Kontraindikationen für NNH-Operation trotz gegebener OP-Indikation: ☐ Nein ☐ Ja
Kontraindikationen für systemische GKS trotz gegebener Indikation: ☐ Nein ☐ Ja
beidseitige Polyposis nasi: ☐ Nein ☐ Ja
Nachweis signifikante Riechstörung: ☐ Nein ☐ Ja
Sniffin' Sticks Score: …………
UPSIT Score: …………
anderer Riechtest: ………… (welcher? ………………………………)
bekanntes Asthma bronchiale: ☐ Nein ☐ Ja
bekannte Neurodermitis: ☐ Nein ☐ Ja
bekanntes ASS-Intoleranz-Syndrom (N-ERD, M. Samter): ☐ Nein ☐ Ja
„Early onset" CRSwNP: ☐ Nein ☐ Ja
Ausschluß andere Ursache: ☐ Nein ☐ Ja
(wie z.B. Mukozelen; Mukoviszidose etc.)

Sonstiges (z.B. relevante Begleiterkrankungen etc.) ……………………………………………………………………………………………
………

Indikation ist gegeben: ☐ **Nein** ☐ **Ja**
Therapieeinleitung mit: ……………………

Datum: …………………………………………… Unterschrift: …………………..……..……………

CRSwNP: Chronische RhinoSinusitis mit Nasenpolypen // GKS: Glukokortikosteroide // UPSIT: University of Pennsylvania Smell Identification Test // N_ERD: Non-steroidal anti-inflammatory Drugs // QoL: Quality of Life

© L. Klimek, Wiesbaden

Abb. 1: Dokumentationsbogen für die Indikationsstellung einer Therapie mit Biologika bei Polyposis nasi (CRSwNP)
